# A Vignette Study to Derive Health-Related Quality-of-Life Weights for Individuals with Steroid Refractory Chronic Graft-versus-Host Disease Receiving Third-Line Therapy in the United Kingdom

**DOI:** 10.36469/001c.125546

**Published:** 2025-02-19

**Authors:** Emma Williams, Luke Skinner, Richard Hudson, Arunesh Sil, Katharina Ecsy, Elisheva Lew, Abdul Jabbar Omar Alsaleh, Elin Gruffydd, Andrew Lloyd, Daniele Avenoso

**Affiliations:** 1 Acaster Lloyd Consulting Ltd, London, UK; 2 Sanofi, Reading, UK; 3 Sanofi, Paris, France; 4 Sanofi, Milan, Italy

**Keywords:** chronic graft-versus-host disease, EQ 5D 5L, EQ-5D-VAS, health state, patient-reported outcomes, quality of life, vignette study

## Abstract

**Background:** Chronic graft-versus-host disease (cGvHD) – a potentially debilitating complication of allogeneic hematopoietic stem cell transplantation – is a rare condition. **Objectives:** This vignette-based study aimed to generate utility values to inform an economic model via an online survey wherein cGvHD health state (HS) vignettes were valued by the general UK population using the EQ-5D-5L and the EQ-5D-visual analog scale (EQ-5D VAS). **Methods:** This non-interventional health-related quality of life (HRQoL) study was conducted in 3 stages across the UK: the development, validation, and valuation of HS vignettes to generate utility values for cGvHD. Four HS for cGvHD were defined based on an economic model partitioning different treatment level responses in patients with cGvHD receiving third-line (3L) therapy (HS1: complete response, HS2: partial response, HS3: lack of response, and HS4: recurrent cGvHD). Draft vignettes were developed for each HS based on 4 previously published GvHD vignettes. The contents of the draft vignettes were reviewed for all aspects of cGvHD symptoms and functional impact and validated through semistructured interviews with 5 clinical experts. The 4 finalized HS vignettes were valued by 300 participants from the UK general population using EQ-5D-5L and EQ-5D VAS. **Results:** Previously published vignettes were used to develop the vignettes for the current study that described GvHD in the context of blood cancer and other rare blood disorders (n = 2 each) and included symptoms, functioning, and quality of life for a patient in the HS. The highest and lowest mean EQ-5D-5L utility scores were observed for HS1 (mean [95% CI]: 0.577 [0.558-0.595]) and HS4 (0.061 [0.034-0.088]), respectively. The EQ-5D-VAS showed the highest and lowest mean utility scores for HS1 (46.8 [44.9-48.6]) and HS4 (25.6 [23.4-27.7]), respectively. **Conclusion:** This study generated utility values for HS vignettes describing symptoms, functioning, and HRQoL for patients with cGvHD receiving 3L therapy. The utility values highlighted a substantial burden of cGvHD and HRQoL impact associated with the treatment response level. However, assessing concordance between utility estimates derived from the vignette-based method in a general population and those from patients with cGvHD is further warranted.

## INTRODUCTION

Chronic graft-versus-host disease (cGvHD) is the main cause of morbidity and mortality following allogeneic hematopoietic stem cell transplantation.^1^ It is characterized by chronic inflammation, abnormal immune regulation, fibrosis, and compromised innate and adaptive cell-mediated immunities. However, despite a standardized pathogenesis, patients may present with a wide range of signs and symptoms.^2^ Chronic GvHD, which manifests in multiple organs and requires prolonged immunosuppressive therapy, occurs in 30% to 70% of patients undergoing allogeneic hematopoietic stem cell transplantation.^3–5^

Chronic GvHD negatively impacts patients’ health-related quality of life (HRQoL), resulting in compromised physical, functional, and psychological well-being and ability to return to work.^6–8^ The magnitude of functional deterioration defines the clinical severity of cGvHD.^9^ Besides a decreased HRQoL, patients with cGvHD may experience anxiety and depression due to several existing symptoms.^1^ Most patients struggle to adapt to the new restrictions resulting from cGvHD in their daily activities.^10^

The management of cGvHD depends on its severity and the extent to which the organs are affected. The fundamental goal of cGvHD treatment is to improve patients’ HRQoL by reducing symptoms and preventing immune-mediated damage and disability, as well as the development of irreversible fibrosis.^11,12^ Presently, glucocorticoids remain the mainstay of cGvHD therapy. Subsequent therapies recommended by the National Health Service include extracorporeal photopheresis, pentostatin, rituximab, andimatinib.^13,14^

An accurate characterization of HRQoL is important because patients with cGvHD often receive multiple lines of therapy and experience an increasing burden of symptoms as the disease progresses.^15,16^ Furthermore, because patients with cGvHD experience significant physical manifestations and functional limitations, understanding their perspective on the treatment response is valuable. The utility values used to evaluate the HRQoL of patients with cGvHD can be derived from generic preference-based measures completed by the patients in clinical trials. The National Institute for Health and Care Excellence (NICE) in the United Kingdom (UK) advocates using utilities derived through the EQ-5D to maximize consistency across appraisals.^17^

Evidence generation around HRQoL for newer treatments can be challenging outside clinical trials; however, approval from health technology assessments is required. An often-used alternative method is a vignette approach, whereby detailed descriptions of HRQoL in each health state are developed and then separately valued by the general population. Vignettes are used to investigate perspectives that may not be accessible through other methods, such as record reviews, interviews, focus groups, diaries, or surveys.^18^ NICE recommendations suggest using vignette methodologies to investigate specific requirements of events or health states for economic models.^19^ The valuation of vignettes is usually performed using the time trade-off method or EQ-5D.^20^ Thus, the present study aimed to estimate the utility values for patients’ health state vignettes associated with cGvHD via an online survey wherein these vignettes were valued by the UK general population using the EQ-5D-5L, which was then scored to generate the utility values. In addition, the present study evaluated the EQ-5D visual analog scale (EQ-5D VAS) using an online survey questionnaire.

## METHODS

### Study Design and Characteristics

This non-interventional HRQoL study was conducted in 3 stages. Stages 1 and 2 involved developing and validating the cGvHD health state vignettes. In Stage 3, the health state vignettes were valued by the UK general population using the EQ-5D-5L and the EQ-5D-visual analog scale (EQ-5D VAS) via an online survey to generate utility values. All study materials were submitted to the WIRB-Copernicus Group (WCG®), a central Institutional Review Board (IRB) in the United States, for ethical review. The study was exempted from ethical review on 25 January 2023.

### Vignette Development

**Figure 1.**
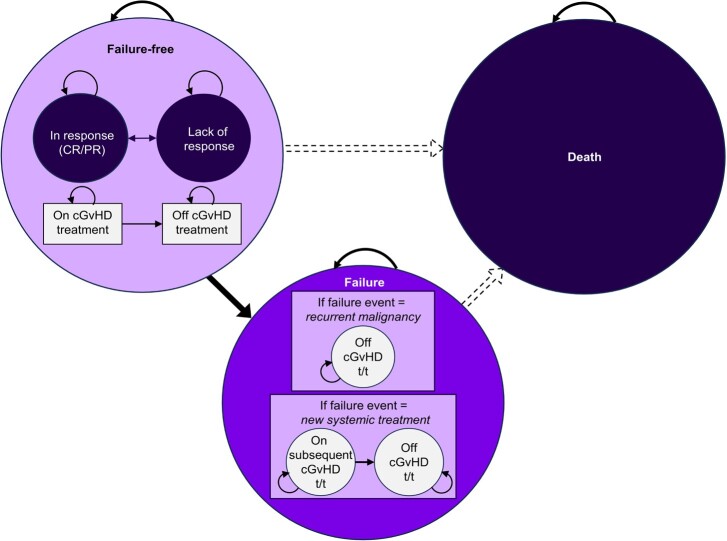
Partitioned Survival Model Structure Abbreviations: cGvHD, chronic graft-versus-host disease; CR, complete response; PR, partial response; t/t, treatment. *Dotted lines* represent transitions between the health states that were not tracked directly.

The health state vignettes were developed to match a cGvHD health state economic model that partitioned patients receiving third-line (3L) therapy across various treatment response levels.^21^ The model structure was partitioned into 3 health states: “failure-free,” “failure,” or “death,” which corresponded to the patient’s post-cGvHD treatment state. The “failure-free” state was stratified as complete response, partial response, or lack of response, while the “treatment failure” state was stratified as relapse of underlying malignancy or change in cGvHD systemic therapy (**Figure 1)**. Additionally, a subclassification of participants who were on and off cGvHD treatment was included in the model. Based on the subclassification, 4 vignettes were developed: health state 1 (failure-free: complete response), health state 2 (failure-free: partial response), health state 3 (failure-free: lack of response), and health state 4 (failure: recurrent cGvHD, after a 3L cGvHD treatment and starting a new systemic treatment).

**Identifying studies including GvHD-related vignettes**: Publicly available GvHD health state vignettes were identified from a literature search and reviewed to develop the draft vignettes for the current study (**Table 1**).^22–25^ Each study described the detailed process of developing the vignettes, including a literature review and interviews with patients and/or clinicians to validate the vignettes. The GvHD health state vignettes identified in the literature were used to develop the draft vignettes describing each cGvHD model state.

**Table 1.** Summary of Included Studies Abbreviations: GvHD, graft-versus-host disease; PSN, peripheral sensory neuropathy.

**Author (Year)**	**Underlying Condition**	**Health States**
Swinburn et al (2015)^22^	Hodgkin lymphoma and systemic anaplastic large cell lymphoma	Stable disease Partial response Complete response Progressive disease Stable disease with B-symptoms Complete response with acute GvHD Complete response with chronic GvHD Complete response with PSN grade I/II Complete response with PSN grade III
Nafees et al (2021)^23^	Hemophagocytic lymphohistiocytosis	Active hemophagocytic lymphohistiocytosis Hemophagocytic lymphohistiocytosis plus neurological symptoms Receiving chemotherapy Undergoing stem cell transplant GvHD Cure End-of-life care
Castejon et al (2018)^24^	Acute myeloid leukemia	Treatment with chemotherapy Consolidation therapy Transplant GvHD Remission Relapse Refractory Functionally cured
Matza et al (2020)^25^	Transfusion-dependent β-thalassemia	Oral chelation Subcutaneous chelation Gene therapy Allogeneic hematopoietic stem cell transplantation Allogeneic hematopoietic stem cell transplantation with acute GvHD Transfusion independent 60% transfusion reduction Chronic GvHD

GvHD, graft-versus-host disease; PSN, peripheral sensory neuropathy.

### Validation of Health State Vignettes

**Interviews with experts**: Interviews were conducted using a semistructured guide to validate and refine the content of the draft health state vignettes with 3 consultant hematologists, 1 consultant nurse, and 1 specialist pharmacist. During the 60-minute semi-structured interviews, the health state vignettes were presented on screen for review. Each bullet point of a vignette was explored in detail, and the clinicians were asked to comment on the accuracy of the descriptions and suggest any changes.

Feedback on the draft health state vignettes was summarized in an Excel spreadsheet. Revisions were made following the first interview; the remaining 4 clinicians reviewed the revised vignettes before they were finalized. After 5 rounds of interviews, the vignettes were finalized after reaching a consensus among the clinical experts. The finalized vignettes are provided in **Appendix 1**.

### Valuation of Health State Vignettes

The finalized vignettes were valued by a representative sample of the UK general population (n = 300) using the EQ-5D-5L and the EQ-5D VAS via an online survey. Participants were recruited via an online recruitment platform (Prolific) to participate in the survey hosted by Qualtrics. No formal sample size calculations were performed as the study did not aim to assess statistical differences between groups. The sample size was based on previous experience and is typical for this type of study.

**Participants**: Eligible participants were aged 18 years or older, living in the UK, and willing and able to provide informed consent to participate in a 20-minute survey. Pre-set quotas were applied through an online recruitment platform (Prolific) to ensure a representative match to the demographic profile of the general UK population (age, sex, ethnicity, and geography).

**Procedures**: An information sheet detailing the study content, procedures, and participants’ rights was provided to eligible participants prior to obtaining their consent via the online survey platform. Participants completed a background questionnaire before completing the main survey. In the main part of the survey, participants provided EQ-5D-5L^26^ and EQ-5D VAS ratings for each health state vignette. The participants were asked to read each vignette and answer a series of questions, imagining that they lived as the individual described. First, the participants were asked to rate the vignettes on a VAS ranging from 0 to 100 (0 = worst health they could imagine; 100 = best health they could imagine). Next, the participants were asked to answer 5 questions about how they imagined their mobility, self-care, usual activities, pain and discomfort, and anxiety and depression that would be affected if they were living as the individual described. The health state vignettes were presented to the participants in a random order.

### Statistical Analysis

The EQ-5D-5L individual responses were mapped into mean EQ-5D-3L utility-based data on the mapping function using scores from Hernandez-Alava et al.^27^ All utility weights were scaled such that 1 represented full health, 0 represented death, and a negative value represented worse than death. The EQ-5D-5L utility data and EQ-5D VAS scores were described using means, 95% confidence intervals (CI), and ranges.

## RESULTS

### Development of Draft Health State Vignettes

Previously published GvHD vignettes from 4 studies were reviewed to develop draft health state vignettes for this study. Two studies described GvHD for blood cancer^22,24^ and the other 2 for rare blood disorders.^23,25^ Two studies described patients with cGvHD,^22,25^ whereas the other 2 did not mention whether GvHD was acute or chronic.^23,24^ None of the studies included patients receiving 3L therapy for GvHD.

All vignettes provided a brief description of the transplant and the success of the treatment. All vignettes described the symptoms and functional problems experienced by the participants owing to GvHD and an increased risk of infection. Dermatological and gastrointestinal symptoms were commonly included. Impacts on daily activities such as washing, dressing, and shopping were also described as well as impacts on social life. The vignettes also included the emotional impact of GvHD, such as depression, anxiety, anger, frustration, and worry about the recurrence of the underlying illness which led to transplantation.

Based on the published evidence, 5 key concepts were included in the draft health state vignettes: a description of the underlying disease and treatment, a description of cGvHD symptoms experienced, the ability to complete daily activities, social impacts, and emotional impacts. Lay language was used to draft the vignettes to ensure they were suitable for valuation by the general population.

### Validation of Draft Health State Vignettes

The accuracy and representativeness of the draft health state vignettes were reviewed by the 5 clinicians, and revisions were made to the contents and wording of the vignettes following feedback. The main changes included the addition of residual symptoms to health state 1 and the replacement of joint stiffness with malnourishment and unintentional weight loss across health states 2 to 4. A new bullet point was added to all health states describing the burden of medication and hospital appointments, as were statements regarding the ability to work, dress, and socialize due to concerns about catching infections. The bullet point about the emotional impact in health state 3 was revised to align more closely with health state 4, and a statement about depression and concerns around the success of further treatment was added to both. Minor updates to wording and additional clarifications were made for accuracy (**Appendix 2**). The clinicians acknowledged that experiences vary between individuals and impacts on HRQoL depend on the extent of organ involvement; however, they agreed that the vignettes were a fair representation of a “typical” patient in each health state.

### Valuation of Health State Vignettes

**Participant characteristics**: A total of 300 participants from the general UK population completed an online survey. Most participants were from England (n = 261, 87.0%), with a mean age of 47.2 years. The detailed characteristics of the participants are presented in **Table 2**.

**Table 2.** Participant Characteristics

**Parameters**	**Total Sample (N = 300)**
Mean (SD) age, years	47.2 (15.8)
Gender (frequency [%])	
Female	146 (48.7)
Male	153 (51.0)
Identifies in another way	1 (0.3)
Location, n (%)	
England	261 (87.0)
Scotland	26 (8.7)
Wales	10 (3.3)
Northern Ireland	3 (1.0)
Living situation, n (%)	
Living with partner/spouse	192 (64.0)
Living alone	61 (20.3)
Living with relatives	35 (11.7)
Other	12 (4.0)
Employment, n (%)	
Full-time	136 (45.3)
Part-time	54 (18.0)
Retired	54 (18.0)
Unemployed	26 (8.7)
Student	19 (6.3)
Other	11 (3.7)
Diagnosis of a chronic illness, n (%)	
No	193 (64.3)
Yes	102 (34.0)
Prefer not to state	5 (1.7)

**EQ-5D-5L**: The EQ-5D-5L utility scores were generated using an online survey (**Appendix 3**). During the online survey, 300 participants rated 4 health states, resulting in 1200 health state valuations. A previously reported mapping function by Hernandez-Alava et al was used to calculate the utility scores.^27^ In addition to the ratings of the 5 domains, the mapping function considered age and gender when estimating utility values. As the mapping function allowed only for binary gender expressions (ie, male or female), the utility scores for 1 participant who responded that they identified in another way could not be calculated. Therefore, 299 participants were included in the final analysis.

**Figure 2.**
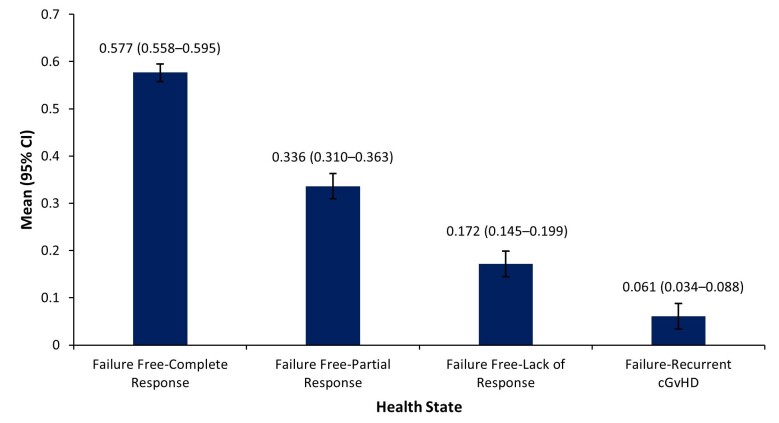
EQ-5D-5L Utility Scores for cGvHD-Related Health States Abbreviations: cGvHD, chronic graft-versus-host disease; CI, confidence interval.

The highest mean utility score (mean [95% CI]: 0.577 [0.558–0.595]) was observed for failure-free: complete response (health state 1), and the lowest mean utility score (0.061 [0.034–0.088]) was observed for failure: recurrent cGvHD (health state 4). The utilities showed a sequential decrease with increasing cGvHD severity (**Figure 2**).

**Figure 3.**
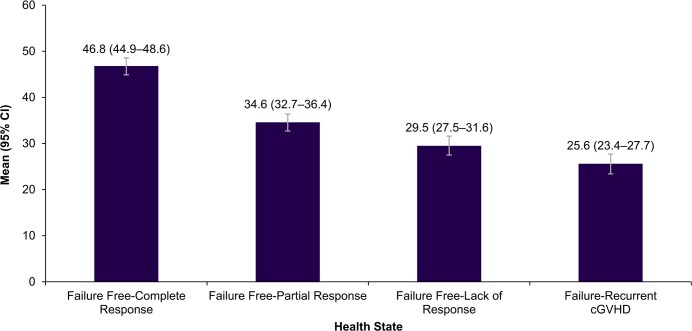
EQ-5D VAS Scores for Health State Vignettes Abbreviations: cGvHD, chronic graft-versus-host disease; CI, confidence interval; EQ-5D VAS, European Quality of Life 5-Dimensional Visual Analog Scale.

**EQ-5D VAS**: All 300 participants rated the vignettes on a scale of 0 to 100. The highest mean VAS score was observed for failure-free: complete response (health state 1: 46.8 [44.9–48.6]), and the lowest mean VAS score was observed for failure: recurrent cGvHD (health state 4: 25.6 [23.4–27.7]). The VAS scores decreased sequentially with increasing cGvHD severity (**Figure 3**).

## DISCUSSION

This study presents utilities for health state vignettes describing different treatment level responses in patients with cGvHD after 2 or more prior therapies. Previously published health state vignettes describing GvHD following stem cell transplantation for hematological conditions were used to develop 4 draft vignettes describing symptoms, functioning, and HRQoL for a typical patient in each health state. The contents of the draft vignettes were validated through interviews with clinical experts. The finalized vignettes were rated by the general UK population using the EQ5D-5L to generate utility values. The highest utility value was observed for failure-free: complete response (health state 1: 0.577 [0.558–0.595]), and the lowest utility value was observed for failure: recurrent cGvHD (health state 4: 0.061 [0.034–0.088]). These utilities substantially impacted the overall HRQoL associated with cGvHD, even in participants who had a complete response to the treatment.

The utility values in the present study ranged from 0.577 to 0.061. Similar values have also been reported in other studies. For example, Castejon et al reported the utility value to be 0.43 (0.36–0.50) for patients with acute myeloid leukemia in the “cGvHD state” among participants in the UK.^24^ Another study reported a “cGvHD state” with a utility value of 0.51.^25^ However, none of the GvHD states in the literature exactly match the states in our study. Given that the present study described 3L treatment (including a treatment failure state), we might expect the values in our study to be lower than those reported in the literature.^24,25^ The EQ-5D-5L utility value for cGvHD for health state 4 (failure: recurrent cGvHD) was 0.061 (0.034–0.088), indicating an HRQoL valued close to that of death. The present study projected the disease impact of cGvHD as per the perception of the general UK population, which may differ from the actual disease impact on the patient. We believe that the differences in patient populations across different studies may contribute to a mismatch in utility values reported across different studies.^24,28^

In a vignette study, the validation of vignettes is crucial for an accurate assessment of health states. The initial draft vignettes were developed based on previously published vignette studies identified via a literature search.^22–25^ The vignettes in the previously published studies were developed through a standard approach wherein initial descriptions were drafted based on evidence from existing literature or consultation with clinical specialists and then validated in qualitative interviews with patients and/or clinical experts. The vignettes used in this study were validated and refined using qualitative interviews with clinical experts. Qualitative data from interviews with clinical experts also provided a rich description of the impact of cGvHD on patients’ lives. The interviews highlighted that cGvHD affected many areas of HRQoL, including physical function, emotional wellbeing, and the ability to participate in daily and social activities. High levels of fatigue, an increased risk of infection, and the unpredictable nature of symptoms result in reduced activity levels and increased time spent at home or in the hospital. Changes in physical function and appearance were reported to be particularly distressing. The qualitative data that informed the vignettes supports the face validity of the utility findings and demonstrated an increasing HRQoL burden with worsening treatment outcomes.

Nonspecific symptoms of cGvHD and an increased risk of infection may lead to reduced activity levels and prolonged hospital stay. Studies have shown a longer hospital stay among patients with infection and those with severe cGvHD.^29,30^ A recent survey showed that almost 33% of patients left their jobs due to cGvHD, and a significant proportion of patients (61.3%) took disability leave.^31^ These data suggest a substantial loss of work productivity in patients with cGvHD. The survey further reported that approximately 30.3% of patients required caregiver support to complete their daily activities.^31^ The study reported that these expenses were due to the requirement of various treatments (41%), inability to resume employment (40%), frequent physician visits (32%), and losing/changing insurance (8%).^32^

Although a few studies have reported the impact of cGvHD on physical functioning, available evidence suggests that the severity of cGvHD is directly associated with its impact on physical functioning and consequent impairment in the patient’s HRQoL.^9,30^ Depression and anxiety are prevalent among patients with cGvHD.^33,34^ The compromised physical functioning has a significant impact on patients’ emotional, psychological, and spiritual well-being.^35,36^ The severity of cGvHD is directly associated with its impact on patients’ HRQoL.^16,30^ Extended hospital stays, increased healthcare costs (such as non-medical costs, including transportation, and medical costs, including prescriptions, copayments, and out-of-pocket costs), long-term immunosuppressive therapy, high financial burden resulting from difficulty paying medical bills, multiple medications, the inability to return to work, frequent physician visits, and losing/changing insurance among patients with severe cGvHD may contribute to the deteriorating HRQoL and the increasing risk of depression and anxiety.^9,32^

Coping strategies used by patients to manage stressful situations may prove impactful in reducing the depression-related symptoms associated with cGvHD.^30^ While cGvHD may affect the mobility of the patient to a considerable extent depending on the disease severity, evidence suggests that increased physical activity, exercise, and emotion-oriented coping strategies are critical to improving the HRQoL of the patients over time.^9,37^ Hence, evaluating patients’ overall HRQoL using tools such as EQ-5D VAS and EQ-5D-5L is helpful for risk stratification and treatment selection according to the extent of impairment in patients’ HRQoL.^38,39^

The draft vignettes were developed from published vignettes based on patient and clinician interviews. The draft health state vignettes were refined and validated through the interviews with clinical experts. However, the vignettes were not reviewed by patients with cGvHD, which is an important limitation of this study. The content of the vignettes may have been limited by potential gaps in the health state descriptions, such as the extent of residual symptoms experienced in the complete response health state. These gaps were overcome through qualitative interviews with clinical experts until a consensus was reached. Another limitation was that the acute or chronic nature of GvHD was not specified in 2 of the studies used to develop the draft health state vignettes, meaning some impacts may not have been relevant to cGvHD specifically. The mapping function used to calculate utilities only allowed for binary expressions of gender (ie, male or female), which could be considered an additional limitation of the present study.^27^

The data generated from interviews with clinical experts provided valuable input regarding the impact of cGvHD on the lives of patients. Patients’ perspectives are extremely important in understanding the impact of cGvHD on several aspects of HRQoL, including physical function, emotional well-being, and ability to continue daily and social activities. These data are important for understanding the impact of treatment on patients with cGvHD and may play a crucial role in providing individualized patient care and informed treatment decisions.

In conclusion, this study generated utility values for health state vignettes describing symptoms, functioning, and HRQoL in participants with cGvHD who had received 3L therapy. These findings highlight the substantial burden of cGvHD and HRQoL benefits associated with treatment response. Further studies considering the wide heterogeneity of the patient population with cGvHD must be conducted to understand its impact on the overall well-being of the participants. The concordance between utility estimates derived from the vignette-based method in the general population and those derived from patients with cGvHD must be further assessed.

### Conflicts of Interest

D.A. was a paid consultant to Sanofi. E.W., E.G., and A.L. were employees of Acaster Lloyd Consulting Ltd, which was paid by Sanofi to undertake this research. K.E., A.S., A.J.O.A., E.L., and R.H. are employees of Sanofi. L.S. was an employee of Sanofi at the time of this study.

### Data Sharing Statement

The data that support the findings of this study are available from the corresponding author upon reasonable request.

## Supplementary Material

Appendix 1: Finalized Vignettes

Appendix 2: Changes to Health State Vignettes

Appendix 3: Online Survey Questions
