# Supplementary material for: A Vignette Study to Derive Health-Related Quality-of-Life Weights for Individuals with Steroid Refractory Chronic Graft-versus-Host Disease Receiving Third-Line Therapy in the United Kingdom
Source: J Health Econ Outcomes Res. 2025 Feb 19;12(1):58–65. doi: 10.36469/001c.125546 (PMC11844253; doi:10.36469/001c.125546)
Supplement: Appendix 1: [file jheor_2025_12_1_125546_263328.pdf]

Appendix 1: Finalized vignettes

Failure-free: complete response (GvHD). On 3L treatment for GvHD

- You have had a transplant to treat a life-threatening illness, which is currently successful.
- You can walk around as normal but experience difficulty walking long distances due to fatigue.
- You have experienced some unwanted residual effects of the transplant:
  - You experienced a **skin rash** with skin thickening and tightening. This has left you with visible scarring and reduced mobility.
  - You have **dry eyes**. This is uncomfortable and requires frequent use of eye drops.
- You have been prescribed medication and must return to hospital for blood tests and routine appointments.
- Currently, you are allowed to be at home, but there is a chance of hospitalization if you catch an infection or your symptoms worsen.
- You can wash and dress yourself and do jobs around the home. You can go shopping but may not be able to complete all daily activities or work because you become tired more easily and worry about catching an infection.
- Your ability to socialize with friends and family is limited due to the symptoms you are experiencing and concerns about catching an infection.
- You experience anxiety and worry about your symptoms returning. You sometimes worry about your illness reoccurring in the future.

Failure-free: partial response (GvHD). On 3L treatment for GvHD

- You have had a transplant to treat a life-threatening illness, which is currently successful.
- You experience difficulty walking any distance due to fatigue. You have experienced some unwanted effects of the transplant:
  - You have **chronic diarrhea**. You experience abdominal pain and often need to go to the toilet urgently.
  - You are malnourished and experience unintentional weight loss due to your high nutritional requirements and chronic diarrhea.
  - You have **dry mouth and dry eyes**. This is uncomfortable and requires frequent use of eye drops and the avoidance of certain foods.
- You have been prescribed medication and must return to hospital at least every 4 weeks for blood tests and to manage your symptoms. Currently, you are allowed to be at home, but there is a chance of hospitalization if you catch an infection or your symptoms worsen.

- You can wash and do jobs around the home. You find dressing difficult due to joint stiffness you experience. You find it difficult to go shopping and are not able to complete daily activities or work because you become tired more easily and worry about catching an infection.
- Your ability to socialize with friends and family is limited due to the symptoms you are experiencing and concerns about catching an infection.
- You experience anxiety because of the unpredictable symptoms you are experiencing. You sometimes worry about your illness reoccurring in the future.

Failure-free: lack of response (GvHD). On 3L treatment for GvHD

- You have had a transplant to treat a life-threatening illness, which is currently successful.
- You experience difficulty walking any distance due to fatigue. You have experienced some unwanted effects of the transplant:
    - You experience a **skin rash** with skin thickening and tightening. The skin rash affects your appearance and mobility.
    - You have **chronic diarrhea**. You experience abdominal pain and often need to go to the toilet urgently.
    - You are malnourished and experience unintentional weight loss due to your high nutritional requirements and chronic diarrhea.
    - You have **dry mouth and dry eyes**. This is uncomfortable and requires frequent use of eye drops and the avoidance of certain foods.
- You have been prescribed a lot of medications and must return to hospital at least once a week for blood tests and to manage your symptoms. Currently, you are allowed to be at home, but you are often hospitalized due to infections and the symptoms you are experiencing.
- You can wash and do minimal jobs around the home with frequent rest breaks. You find dressing difficult due to joint stiffness you experience. You are not able to go shopping, go to work, or complete daily activities because you become extremely tired easily and worry about catching an infection.
- Your ability to socialize with friends and family is limited due to the symptoms you are experiencing and concerns about catching an infection.
- You experience anxiety and depression because you are aware that you have not responded to treatment. You are concerned about the necessity and success of further treatment and the prospect of your symptoms worsening. You sometimes feel worried about dying if your future treatment is not successful.

Failure: recurrent GvHD after 3L treatment for GvHD

- You have had a transplant to treat a life-threatening illness, which is currently successful.
- You experience difficulty walking any distance due to fatigue. You have experienced some unwanted effects of the transplant:
  - You experience a **skin rash** with skin thickening and tightening. The skin rash affects your appearance and mobility.
  - You have **chronic diarrhea**. You experience abdominal pain and often need to go to the toilet urgently.
  - You are malnourished and experience unintentional weight loss due to your high nutritional requirements and chronic diarrhea.
  - You have **dry mouth and dry eyes**. This is uncomfortable and requires frequent use of eye drops and the avoidance of certain foods.
- You have been prescribed a lot of medications and must return to hospital at least twice a week for blood tests and to manage your symptoms. Currently, you spend most of your time in hospital due to catching infections and the symptoms you are experiencing.
- You may be able to wash yourself but find dressing difficult due to joint stiffness you experience. You are not able to do jobs around the home, go shopping, go to work, or complete daily activities as you spend most of your time resting due to extreme fatigue or in hospital.
- Your ability to socialize with friends and family is limited due to the symptoms you are experiencing and concerns about catching an infection.
- You experience anxiety and depression because you are aware that your treatment has not worked. You are concerned about the necessity and success of further treatment and the prospect of your symptoms worsening. You sometimes feel worried about dying if your future treatment is not successful.

GvHD, graft-versus-host disease; 3L, third line.
