# Supplementary material for: A Vignette Study to Derive Health-Related Quality-of-Life Weights for Individuals with Steroid Refractory Chronic Graft-versus-Host Disease Receiving Third-Line Therapy in the United Kingdom
Source: J Health Econ Outcomes Res. 2025 Feb 19;12(1):58–65. doi: 10.36469/001c.125546 (PMC11844253; doi:10.36469/001c.125546)
Supplement: Appendix 2: [file jheor_2025_12_1_125546_263329.pdf]

Appendix 2: Changes to health state vignettes

Concept	Original text	Revised text
**Failure-free: complete response (GvHD). On 3L treatment for GvHD**		
Overview of condition and treatment	You have undergone treatment for a potentially life-threatening illness, which is currently successful. You have an increased risk of the disease reoccurring in the future.	You have had a transplant to treat a life-threatening illness, which is currently successful.
Symptoms and functional impacts	You are able to walk around as normal, but experience difficulty in walking long distances due to fatigue.	You are able to walk around as normal, but experience difficulty in walking long distances due to fatigue. You have experienced some unwanted residual effects of the transplant:<br>• You experienced a skin rash with skin thickening and tightening. This has left you with visible scarring and reduced mobility.<br>• You have dry eyes. This is uncomfortable and requires frequent use of eye drops.
Medication and Appointments		You have been prescribed medication and have to return to hospital for blood tests and routine appointments. Currently, you are allowed to be at home, but there is a chance of hospitalisation if you catch an infection, or your symptoms worsen.
Daily activities	You are able to wash and dress yourself and do jobs around the home. You are able to go shopping but you may not be able to complete all daily activities because you become tired more easily and worry about catching an infection.	You are able to wash and dress yourself and do jobs around the home. You are able to go shopping but you may not be able to complete all daily activities or work because you become tired more easily and worry about catching an infection.

Concept	Original text	Revised text
Social impact	Your ability to socialise with friends and family is limited due to your illness.	Your ability to socialise with friends and family is limited due to the symptoms you are experiencing and concerns about catching an infection.
Emotional impact	You experience anxiety sometimes and worry about your illness reoccurring in the future.	You experience anxiety and worry about your symptoms returning. You sometimes worry about your illness reoccurring in the future.
**Failure free – Partial response (GvHD). On 3L treatment for GvHD**		
Overview of condition and treatment	You have undergone treatment for a potentially life-threatening illness, which is currently successful. You have an increased risk of the disease reoccurring in the future.	You have had a transplant to treat a life-threatening illness, which is currently successful.
Symptoms and functional impacts	You experience difficulty in walking any distance due to fatigue and very stiff joints. You have experienced some unwanted effects of treatment:<br>• You have chronic diarrhoea, unintentional weight loss, and abdominal pain.<br>• You experience stiffening of your joints. This is painful and persistent, and it causes you to limit your physical activity.<br>• You have dry mouth and dry eyes. This can be uncomfortable.	You experience difficulty in walking any distance due to fatigue. You have experienced some unwanted effects of the transplant:<br>• You have chronic diarrhoea. You experience abdominal pain and often need to go to the toilet urgently.<br>• You are malnourished and experience unintentional weight loss due to your high nutritional requirements and chronic diarrhoea.<br>• You have dry mouth and dry eyes. This is uncomfortable and requires frequent use of eye drops and the avoidance of certain foods.
Medication and Appointments		You have been prescribed medication and have to return to hospital at least every four weeks for blood tests and to manage your symptoms. Currently, you are allowed to

Concept	Original text	Revised text
		be at home, but there is a chance of hospitalisation if you catch an infection, or your symptoms worsen.
Daily activities	You are able to wash and dress yourself and do jobs around the home. You are able to go shopping but you are not able to complete daily activities because you become tired more easily and worry about catching an infection.	You are able to wash and do jobs around the home. You find dressing difficult due to joint stiffness you experience. You find it difficult to go shopping and are not able to complete daily activities or work because you become tired more easily and worry about catching an infection.
Social impact	Your ability to socialise with friends and family is limited due to your illness.	Your ability to socialise with friends and family is limited due to the symptoms you are experiencing and concerns about catching an infection.
Emotional Impact	You experience anxiety because of the symptoms you are experiencing, and you sometimes worry about your illness reoccurring in the future.	You experience anxiety because of the unpredictable symptoms you are experiencing. You sometimes worry about your illness reoccurring in the future.
**Failure free – Lack of response (GvHD). On 3L treatment for GvHD**		
Overview of condition and treatment	You have undergone treatment for a potentially life-threatening illness, which is currently successful.<br>You have an increased risk of the disease reoccurring in the future.	You have had a transplant to treat a life-threatening illness, which is currently successful.
Symptoms and functional impacts	You experience difficulty in walking any distance due to fatigue and very stiff joints. You have experienced some unwanted effects of treatment:<br>• You experience a skin rash with skin thickening and tightening. The skin rash affects your appearance.<br>• You have chronic diarrhoea, unintentional weight loss, and abdominal pain.	You experience difficulty in walking any distance due to fatigue.<br>You have experienced some unwanted effects of the transplant:<br>• You experience a skin rash with skin thickening and tightening. The skin rash affects your appearance and mobility.

Concept	Original text	Revised text
	• You experience stiffening of your joints. This is painful and persistent, and it causes you to limit your physical activity.<br>• You have dry mouth and dry eyes. This can be uncomfortable.	• You have chronic diarrhoea. You experience abdominal pain and often need to go to the toilet urgently.<br>• You are malnourished and experience unintentional weight loss due to your high nutritional requirements and chronic diarrhoea.<br>• You have dry mouth and dry eyes. This is uncomfortable and requires frequent use of eye drops and the avoidance of certain foods.
Medication and Appointments		You have been prescribed a lot of medication and have to return to hospital at least once a week for blood tests and to manage your symptoms. Currently, you are allowed to be at home, but are often hospitalised due to catching infections and the symptoms you are experiencing.
Daily activities	You are able to wash and dress yourself and do jobs around the home. You are able to go shopping but you are not able to complete daily activities because you become tired more easily and worry about catching an infection.	You are able to wash and do minimal jobs around the home with frequent rest breaks. You find dressing difficult due to joint stiffness you experience. You are not able to go shopping, go to work or complete daily activities because you become tired extremely easily and worry about catching an infection.
Social impact	Your ability to socialise with friends and family is limited due to your illness.	Your ability to socialise with friends and family is limited due to the symptoms you are experiencing and concerns about catching an infection.
Emotional Impact	You experience anxiety because of the symptoms you are experiencing, and you sometimes worry about your illness reoccurring in the future.	You experience anxiety and depression because you are aware that you have not responded to treatment. You are concerned about the necessity and success of further

Concept	Original text	Revised text
		treatment and the prospect of your symptoms worsening.<br>You sometimes feel worried about dying if your future treatment is not successful.
**Failure – recurrent GvHD after 3L treatment for GvHD**		
Overview of condition and treatment	You have undergone treatment for a potentially life-threatening illness, which is currently successful. You have an increased risk of the disease reoccurring in the future.	You have had a transplant to treat a life-threatening illness, which is currently successful.
Symptoms and functional impacts	You experience difficulty in walking any distance due to fatigue and very stiff joints. You have experienced some unwanted effects of treatment:<br>• You experience a skin rash with skin thickening and tightening. The skin rash affects your appearance.<br>• You have chronic diarrhoea, unintentional weight loss, and abdominal pain.<br>• You experience stiffening of your joints. This is painful and persistent, and it causes you to limit your physical activity.<br>• You have dry mouth and dry eyes. This can be uncomfortable.	You experience difficulty in walking any distance due to fatigue. You have experienced some unwanted effects of the transplant:<br>• You experience a skin rash with skin thickening and tightening. The skin rash affects your appearance and mobility.<br>• You have chronic diarrhoea. You experience abdominal pain and often need to go to the toilet urgently.<br>• You are malnourished and experience unintentional weight loss due to your high nutritional requirements and chronic diarrhoea.<br>• You have dry mouth and dry eyes. This is uncomfortable and requires frequent use of eye drops and the avoidance of certain foods.
Medication and Appointments		You have been prescribed a lot of medication and have to return to hospital at least twice a week for blood tests and to manage your symptoms. Currently, you spend

Concept	Original text	Revised text
		most of your time in hospital due to catching infections and the symptoms you are experiencing.
Daily activities	You are able to wash and dress yourself and do jobs around the home. You are able to complete light daily activities at home although you become tired more easily.	You may be able to wash yourself but find dressing difficult due to joint stiffness you experience. You are not able to do jobs around the home, go shopping, go to work or complete daily activities as you spend most of your time resting due to extreme fatigue or in hospital.
Social impact	Your ability to socialise with friends and family is limited due to your illness.	Your ability to socialise with friends and family is limited due to the symptoms you are experiencing and concerns about catching an infection.
Emotional Impact	You experience anxiety because you are aware that the treatments you have received would have cured the majority of people. You are concerned about the necessity for further treatment and the prospect of your disease worsening. You sometimes feel worried about dying if your future treatment is not successful.	You experience anxiety and depression because you are aware that your treatment has not worked. You are concerned about the necessity and success of further treatment and the prospect of your symptoms worsening. You sometimes feel worried about dying if your future treatment is not successful.
