# Supplementary material for: A Vignette Study to Derive Health-Related Quality-of-Life Weights for Individuals with Steroid Refractory Chronic Graft-versus-Host Disease Receiving Third-Line Therapy in the United Kingdom
Source: J Health Econ Outcomes Res. 2025 Feb 19;12(1):58–65. doi: 10.36469/001c.125546 (PMC11844253; doi:10.36469/001c.125546)
Supplement: Appendix 3: [file jheor_2025_12_1_125546_263330.pdf]

Appendix 3: Online Survey Questions

Survey introduction

In the main part of the survey, you will be asked to read **four** different descriptions in turn that briefly outline a state of health. We would like you to imagine that you are living as the individual described in the state of health and think about how your life would be impacted. **Please spend a few minutes reading each description in detail. Although some descriptions may seem similar, there are differences between all the descriptions. Please pay attention to these differences.**

After reading each description you will be asked to complete a series of questions imagining that you are living as the individual described in the state of health. After you have answered the questions, you will be shown the next description and asked the same set of questions. This may seem repetitive, but please remember that you are being asked the same set of questions about **different descriptions.**

Health state valuation [REPEAT FOR EACH HEALTH STATE VIGNETTE]

On the next page you will be shown one of the four descriptions.

Please click the arrow when you are ready to proceed.

Please read this **new** description carefully and imagine that you are living as the individual described. Please think about how your life would be impacted. You do not need to memorise the description as you will still be able to see it during the next part of the survey.

[INSERT RELEVANT HEALTH STATE VIGNETTE]

Imagining that you are living as the individual described, we would like to know how good or bad your health is. The line below is numbered from 0 to 100. 100 means the best health you can imagine. 0 means the worst health you can imagine.

1. Imagining that you are living as the individual described, please write a number from 0 to 100 to show how good or bad your health is. **[numeric forced response from 0 to 100]**

Please see below for a reminder of the description.

[INSERT RELEVANT HEALTH STATE VIGNETTE]

Please imagine that are you living the same hypothetical life described and under each heading, tick the ONE box that best describes your health.

Please find a reminder of the description at the bottom of the page.

2. MOBILITY **[single forced response]**

- ☐ I have no problems in walking about
- ☐ I have slight problems in walking about
- ☐ I have moderate problems in walking about
- ☐ I have severe problems in walking about
- ☐ I am unable to walk about

3. SELF-CARE **[single forced response]**

- ☐ I have no problems with washing or dressing myself
- ☐ I have slight problems with washing or dressing myself
- ☐ I have moderate problems with washing or dressing myself
- ☐ I have severe problems with washing or dressing myself
- ☐ I am unable to wash or dress myself

4. USUAL ACTIVITIES (e.g. work, study, housework, family or leisure activities). **[single forced response]**

- ☐ I have no problems doing my usual activities
- ☐ I have slight problems doing my usual activities
- ☐ I have moderate problems doing my usual activities
- ☐ I have severe problems doing my usual activities
- ☐ I am unable to do my usual activities

5. PAIN / DISCOMFORT **[single forced response]**

- ☐ I have no pain or discomfort
- ☐ I have slight pain or discomfort
- ☐ I have moderate pain or discomfort
- ☐ I have severe pain or discomfort
- ☐ I have extreme pain or discomfort

6. ANXIETY / DEPRESSION **[single forced response]**

- ☐ I am not anxious or depressed
- ☐ I am slightly anxious or depressed
- ☐ I am moderately anxious or depressed
- ☐ I am severely anxious or depressed
- ☐ I am extremely anxious or depressed

[INSERT RELEVANT HEALTH STATE VIGNETTE]

Feedback

Thank you for answering those questions. You have now reached the end of the survey. Before you return to Prolific, please let us know if you have any comments about the survey. **[free text response]**

End of survey

Thank you for taking part in this survey. If you have any questions or concerns about this research, please contact the research team via Prolific.
